# DEPRESSIVE SYMPTOMS ACROSS THE LIFESPAN: DIFFERENCES IN SYMPTOM CRITERIA ENDORSEMENT

**DOI:** 10.1093/geroni/igae098.2669

**Published:** 2024-12-31

**Authors:** Teresa Walker, Brenna Renn

**Affiliations:** University of Nevada Las Vegas, Las Vegas, Nevada, United States; University of Nevada Las Vegas, Las Vegas, Nevada, United States

## Abstract

Scientific literature and clinical wisdom often cite older adults as more likely to endorse somatic rather than mood symptoms of depression compared to younger people. However, this is based on older data and needs updating with demographic shifts. As many older adults with clinically significant symptoms of depression go undiagnosed and untreated, with deleterious effects on health outcomes and quality of life, identifying and treating depression is necessary to provide quality healthcare for older adults. Thus, the purpose of the current study was to investigate differences in endorsements of depressive symptoms across age groups with recent data from the National Health and Nutrition Examination Survey 2017-2020 Pre-Pandemic dataset. Participants (N = 8299) were categorized into young adults (18-24 years), middle adults (25-55 years), young-older adults (56-75 years), and older adults (76+ years). Responses to items on the Patient Health Questionnaire (PHQ-9) were dichotomized into meeting criteria for a depressive episode (vs. not). Chi-squared tests of independence revealed significant differences across some age groups for symptoms including sleep disturbance, feelings of worthlessness/guilt, and suicidal ideation. Young-older adults were the only age group to significantly differ from at least one other age group on all three of these items. Of particular note, older adults did not significantly differ from other age groups on any items, contradicting previous findings that older adults report more somatic symptoms than their younger counterparts. These findings contribute nuance to understanding depressive symptom presentation across the lifespan to guide clinical assessment of older adults.

